# Self-Standing 3D-Printed PEGDA–PANIs Electroconductive Hydrogel Composites for pH Monitoring

**DOI:** 10.3390/gels9100784

**Published:** 2023-09-26

**Authors:** Rocco Carcione, Francesca Pescosolido, Luca Montaina, Francesco Toschi, Silvia Orlanducci, Emanuela Tamburri, Silvia Battistoni

**Affiliations:** 1 Consiglio Nazionale delle Ricerche, Institute of Materials for Electronics and Magnetism (CNR-IMEM), Parco Area delle Scienze 37A, 43124 Parma, Italy; rocco.carcione@imem.cnr.it; 2 Dipartimento di Scienze e Tecnologie Chimiche & UdR INSTM di Roma, Università degli Studi di Roma “Tor Vergata”—Via della Ricerca Scientifica, 00133 Rome, Italy; francesca.pescosolido@alumni.uniroma2.eu (F.P.); luca.montaina@alumni.uniroma2.eu (L.M.); silvia.orlanducci@uniroma2.it (S.O.); 3 Centro di Ricerca Interdipartimentale di Medicina Rigenerativa (CIMER), Università degli Studi di Roma “Tor Vergata”, Via Montpellier 1, 00133 Rome, Italy; 4 Istituto di Struttura della Materia—CNR (ISM-CNR) & EuroFEL Support Laboratory (EFSL), 00015 Monterotondo Scalo, Italy; francesco.toschi@cnr.it

**Keywords:** conductive polymers, sulfonated polyaniline, 3D printing, additive manufacturing, flexible electronics, pH monitoring, electrically conductive hydrogels

## Abstract

Additive manufacturing (AM), or 3D printing processes, is introducing new possibilities in electronic, biomedical, sensor-designing, and wearable technologies. In this context, the present work focuses on the development of flexible 3D-printed polyethylene glycol diacrylate (PEGDA)- sulfonated polyaniline (PANIs) electrically conductive hydrogels (ECHs) for pH-monitoring applications. PEGDA platforms are 3D printed by a stereolithography (SLA) approach. Here, we report the successful realization of PEGDA–PANIs electroconductive hydrogel (ECH) composites produced by an in situ chemical oxidative co-polymerization of aniline (ANI) and aniline 2-sulfonic acid (ANIs) monomers at a 1:1 equimolar ratio in acidic medium. The morphological and functional properties of PEGDA–PANIs are compared to those of PEGDA–PANI composites by coupling SEM, swelling degree, I–V, and electro–chemo–mechanical analyses. The differences are discussed as a function of morphological, structural, and charge transfer/transport properties of the respective PANIs and PANI filler. Our investigation showed that the electrochemical activity of PANIs allows for the exploitation of the PEGDA–PANIs composite as an electrode material for pH monitoring in a linear range compatible with that of most biofluids. This feature, combined with the superior electromechanical behavior, swelling capacity, and water retention properties, makes PEGDA–PANIs hydrogel a promising active material for developing advanced biomedical, soft tissue, and biocompatible electronic applications.

## 1. Introduction

Electrically conductive hydrogels (ECHs) belong to an emerging class of materials produced by the incorporation of electroconductive elements or compounds into hydrogel networks [1,2,3]. The capability of hydrogels to absorb and retain large amounts of water while maintaining their structural integrity, combined with the electrical conductivity of the fillers, makes ECHs particularly attractive for a wide range of advanced technologies, such as smart bioelectronics, soft electrodes, drug delivery, tissue-engineered scaffolds, and sensing and wearable applications [4,5,6,7,8,9]. Unfortunately, traditional methods for producing 3D architecture can limit control over the structure and properties of hydrogel materials, leading to variations in mechanical strength, swelling behavior, porosity, and other important characteristics of these systems. However, the 3D printability evidenced for some of them has been exploited in additive manufacturing (AM) technologies, which are revolutionizing material preparation and processing methods [10,11,12]. In fact, AM has proven to be able to produce intricate, customizable, and functional structures and is easily integrated into electronic, biomedical, and sensor devices [13,14,15,16,17,18,19,20,21,22,23,24,25,26,27,28,29]. Specifically, vat polymerization through stereolithography (SLA) can be considered one of the most convenient AM approaches. The process is based on the selective curing of a photocurable ink induced by UV light radiation [30]. The SLA 3D printers differ from each other in how the printed layers are processed, i.e., with a controlled laser beam, a digital projector, or LEDs and an LCD mask (generally known as MSLA). The use of light to cure ink enables a fine control of surface quality and finishes by guaranteeing high resolution, accuracy, and sharp details [31].

Among the non-toxic biodegradable hydrogels suitable for 3D printing, the choice of the polyethylene glycol diacrylate (PEGDA) polymer is often the most common thanks to its water processability and rheological and mechanical properties, as well as its resistance to heat, acids, bases, and salts [32]. In the scenario of the possible fillers to be incorporated within the PEGDA matrix to produce ECHs, conductive polymers (CPs) offer an exciting alternative to metallic and carbon-based nanoparticles [2,3,13,23,33,34]. In particular, polyaniline (PANI) has the advantage of combining electrical, electrochemical, mechanical, and peculiar optical properties with low cost, easy processability, and stability in air. These features can be finely tuned by varying synthesis methods, catalysts, reaction conditions, and doping strategies, as well as by employing properly functionalized monomers [35,36,37,38]. Regarding the latter aspect, we can emphasize how the presence of a sulfonic acid group (-SO_3_H) covalently bonded to the aniline (ANI) ring induces a permanent hydrophilic modification and self-doping of the related sulfonated polyaniline (PANIs) polymer. Although the increase in the sulfonation grade was found to lower the charge transport properties of PANIs chains at low pH levels, the sulfonic functional groups on the polymeric backbone allow for greater water solubility and proved to extend the electrical and electrochemical activity until pH 7 [39,40,41,42,43].

The first synthesis of PANIs was reported by Yue et al., who used the emerald base form of PANI as a starting material and subsequently sulfonated it with fuming sulfonic acid [43]. Then, Yang et al. [44] reported the synthesis and characterization of PANIs by the oxidative co-polymerization of ANI and aniline-2-sulfonic acid (ANIs) monomers promoted by ammonium persulfate (APS) as an oxidant in 1 M HCl. This one-pot strategy is the more straightforward method to synthesize PANIs in the electrically conductive emeraldine salt (ES) form [39,40,42,43,45,46]. In fact, the synthesis of PANIs by using equimolar amounts of ANI and ANIs monomers allows for the growing of PANIs that are both electrically and electrochemically active within the range of physiological pH levels.

Analogously to PANI, the exploitation of PANIs as an active material for organic electronics can be accomplished by means of various deposition methods, such as spraying and drop casting [47], dip coating [48], spin coating [49], and Langmuir Blodgett/Schaeffer techniques [50,51]. Recently, we investigated different methodologies to produce 3D PEGDA–PANI composites, including an innovative approach that involves aniline photopolymerization during an SLA printing procedure [13,23]. Another promising strategy is the in situ polymerization of CPs precursors within already 3D-printed PEGDA items. Considering that fine customization becomes crucial for specific applications, this approach is often essential to finalize the structural integrity and maintain the properties of the 3D-printed objects. In this view, the present work is devoted to combining SLA with the in situ polymerization of PANIs precursors for fabricating self-standing PEGDA–PANIs ECHs with electro–chemo–mechanical properties suitable for pH monitoring.

Recent studies have already focused on PANI-based composites for sensing applications [52,53,54,55,56,57]. However, the activity of the polymer phase was generally supported by the presence of nanoparticles [53,54,56] or metal or carbon electrodes [52,57]. The novelty of the present research relies on the production of a fully 3D-printed polymeric-active material with pH-responsive properties tunable in the 2–7 pH range. Specifically, the objective of the present research is to set effective chemical protocols for the production of self-standing 3D-printed PEGDA–PANIs electroconductive hydrogel composites for pH monitoring. This goal was achieved by exploiting the in situ chemical oxidative co-polymerization of ANI and ANIs monomers within the meshes of PEGDA-printed structures, previously soaked in a precursor solution. IR, Raman spectroscopy, XRD, SEM, I-V, cyclic voltammetry (CV), electrochemical impedance spectroscopy (EIS), and electromechanical tests were performed to study the structure, morphology, and functionality of the produced composites. Swelling degree and deswelling kinetics were investigated to disclose whether the presence of CP filler could maintain the absorption and water-retaining capabilities of PEGDA support.

The obtained preliminary results demonstrate that produced PEGDA–PANIs ECHs exhibit a linear response for monitoring pH across the clinically relevant range for most biofluids, such as vaginal fluid [58], sweat [59], gastroesophageal reflux [60], and urine [61]. In this view, the novelty and the impact of the present research are reflected in the settling of appropriate and simple methodologies to print PEGDA–PANIs ECHs in 3D-shaped and intricate structures, showing charge transport and electromechanical properties suitable for a variety of possible electroanalytical applications at physiological pH environments.

## 2. Results and Discussion

### 2.1. PANIs and PANI Characterizations

**Figure 1:**
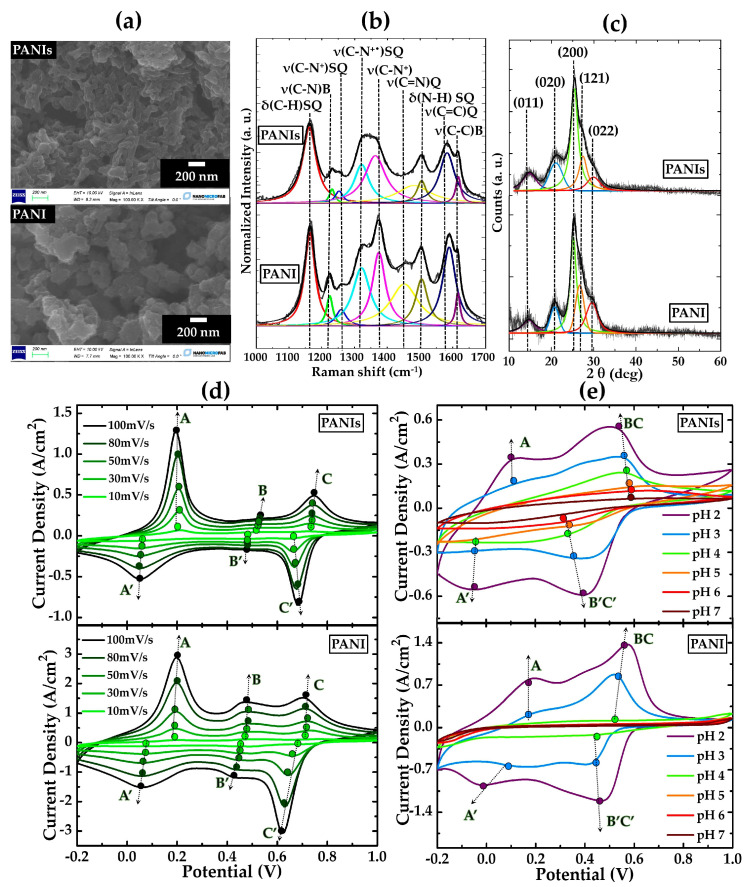
(**a**) SEM images obtained at high magnification for PANIs and PANI; deconvolved (**b**) Raman spectra and (**c**) XRD patterns along with signal’s attribution for PANIs and PANI; (**d**) CV curves at 10, 30, 50, 80, and 100 mV/s for PANIs and PANI in 1 M HCl; (**e**) CV curves at 100 mV/s in 0.1 M NaCl solutions with pH ranging from 2 to 7 for PANIs and PANI.

Figure 1 shows the morphological, structural, and electrochemical characterizations of PANIs and PANI reference samples.

It can be observed that, while PANI shows a granular agglomerate texture, PANIs is constituted by short nanofibers (Figure 1a). This different morphology can be related to the presence of the sulfonic groups on PANIs chains, which are reasonably responsible for a series of effects such as solvation, steric hindrance, and static repulsion. These phenomena are thus expected to reduce the interactions among the polymeric backbones, resulting in the formation of elongated aggregates [62,63].

The structural features of the two polymeric systems were evaluated by means of FT–IR, Raman spectroscopy, and XRD techniques. As pointed out by Raman spectra (Figure 1b and Note S1), the molecular structure of both the samples shows a series of features typical of semiquinonoid (SQ) structures, which are commonly related to PANI emeraldine salt (ES) [64,65,66]. The FT–IR analysis (Figure S1) evidences the presence of sulfonic groups on the PANIs backbones regardless of the use of APS. For more details, a comprehensive attribution of FT–IR and Raman signals can be found in Supporting Information.

The study of crystallinity by XRD allowed for further highlighting the effects of the main chains’ sulfonation (Figure 1c) [67]. In particular, the calculation of the average crystallite size of the produced samples (for details see Note S2) provided a value of 40 and 47 nm, respectively, for PANIs and PANI. Once again, the lower PANIs grain size can be imputed to sulfonic groups that, increasing the steric interactions among polymer chains, reasonably cause a lowering of the chain stacking [68]. Accordingly, conductivity (σ) values in the order of 400 and 40 S/m were found for PANI and PANIs, respectively. In this view, the lower σ value of a PANIs sample can be due to the rise of the energy required for interchain charge transport [69,70,71].

As a consequence of the different electrical conductivities, current density values in the PANI CV curves are almost double than those observed in PANIs cyclic voltammograms (Figure 1d). Nevertheless, PANI and PANIs show rather similar current/potential profiles, which are featured by three pairs of quasi-reversible peaks (AA’, BB’, and CC’). The most common attribution for these peaks is related to the transitions between leucoemeraldine and emeraldine (L–E) (AA’) and between emeraldine and pernigraniline (E–P) (CC’) oxidation forms, as well as to the formation of p-benzoquinone and hydroquinone species, which are intermediate structures in the conversion from the partially oxidized to the fully oxidized configuration (BB’) [72,73,74]. However, a possible contribution of hydrogen adsorption/desorption processes with the polymer phases cannot be excluded (AA’) [75,76]. In addition, a shift of anodic and cathodic peaks towards greater and lower potentials with the sweep rate increasing was also found (black dashed arrows in CV curves), respectively, for PANI and PANIs samples. Such an electrochemical behavior can be explained by redox processes involving the polymer backbone occurring under a diffusion regimen.

Different electrochemical features were instead found in NaCl solutions buffered to a pH greater than 3 (Figure 1e). With the pH rise, we observe that the B and C oxidation and B′ and C′ reduction signals coalesce in a single broad anodic (BC) and cathodic (B’C’) peak, respectively, while the AA’ pair progressively vanishes. Considering that the latter can be attributable to reactions involving hydrogen species and/or emeraldine formation, its disappearance can be explained by the hydronium ions’ concentration decreasing and/or with a deprotonation effect given by the pH increasing [72,74,77]. Finally, an extension of the electrochemical activity in a broader pH range was found only for PANIs samples. This capability can be due to the presence of sulfonic groups on PANIs chains, which can ionize in aqueous media according to the reaction (1) [78]:

(1) $$R-{SO}_{3}H⇄{RSO}_{3}^{-}+H^{+}$$

where the hydrogen ions are expected to be transferred to the polymer backbone, thus supporting its redox reactions until a neutral pH. This peculiar electrochemical performance modulation within the 2–7 pH range undoubtedly provides an added value to PANIs systems since they extend the use of such material in physiological environments.

### 2.2. PEGDA–PANIs and PEGDA–PANI Composites

#### 2.2.1. Morphological and Structural Characterization

**Figure 2:**
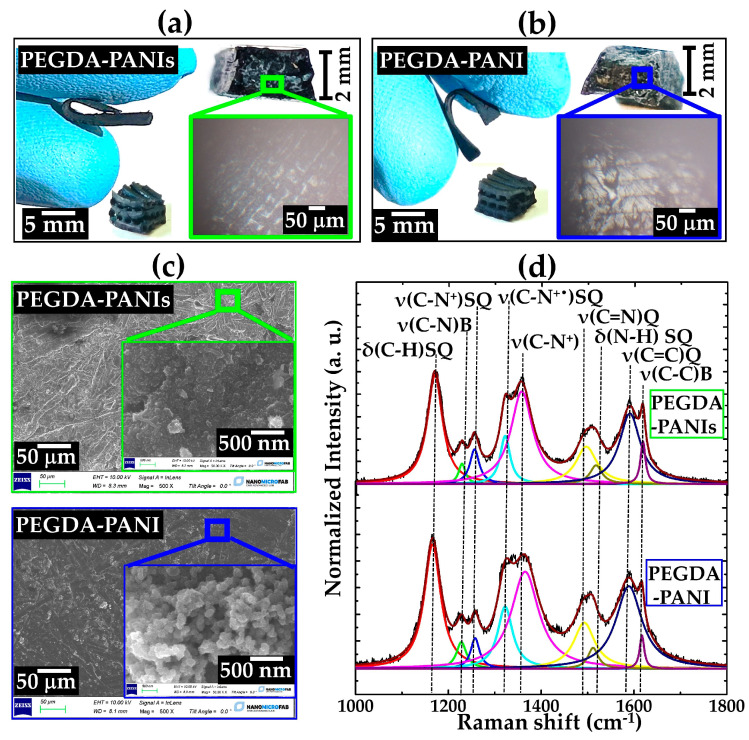
(**a**,**b**) Photos showing the aspect and the flexibility of the 3D-printed PEGDA–PANIs and PEGDA–PANI objects along with their respective cross-section optical microscope photos; (**c**) SEM images and (**d**) Deconvolved and normalized Raman spectra with signals’ attribution for PEGDA–PANIs and PEGDA–PANI samples.

In Figure 2, the photos showing typical 3D PEGDA–PANIs and PEGDA–PANI items are reported along with the respective optical microscope photos, SEM images, and deconvolved Raman spectra.

The settled protocol for in situ polymerization enables the production of PEGDA–PANIs and PEGDA–PANI composites with both simple (parallelepiped) and complex (woodpile) architectures, both characterized by a remarkable and uniform dark green coloration typical of ES configuration (Figure 2a,b). The cross-section photos shown in the inset highlight both the PEGDA multilayer texture and the uniform permeation of the CP filler in the whole structure, evidencing the formation of homogeneous composite materials. Moreover, the samples prove to be endowed with marked flexibility, coming from the PEGDA matrix, and completely preserved after the CPs’ growth. In particular, the bending radius can be estimated at 3 mm for both composites. Interestingly, these values are in line with some peculiarly curved surfaces of human skin, such as the nose, ear, and articular surfaces [79], indicating the potential use of the produced materials in applications requiring physical body contact. Furthermore, SEM images allow for the focus on the surface finish of the printed objects, which highlights the uniform coverage by PANI and PANIs that, in turn, show a tubular and globular morphology, respectively (Figure 2c).

In line with the dark-green coloring, the Raman spectra of PEGDA–PANIs and PEGDA–PANI show the typical features of the delocalized polaronic structures related to the ES form (Figure 2d). Therefore, the presence of both semiquinonoid radical and benzenoid signals in composites’ spectra indicates that the in situ polymerization also enables the formation of partially protonated chains analogously to what was found for PANIs and PANI samples. The protonation percentage of PANI and PANIs fillers was evaluated by using Equation (2) [80]:

(2) $$protonation\;percentage=\frac{A_{1320}}{A_{1225}+A_{1320}}\cdot 100\;$$

where *A*_1320_ and *A*_1225_ are the integrated areas of the peaks located at 1320 and 1225 cm^−1^, respectively, attributable to the stretching vibration of the polaronic and benzenoid C–N bonds. Values of 78% and 77% obtained for PEGDA–PANI and PEGDA–PANIs indicate a high protonation level of the main chains for both fillers and independent of sulfonation.

#### 2.2.2. Swelling Degree and Water Retention Analyses

**Figure 3:**
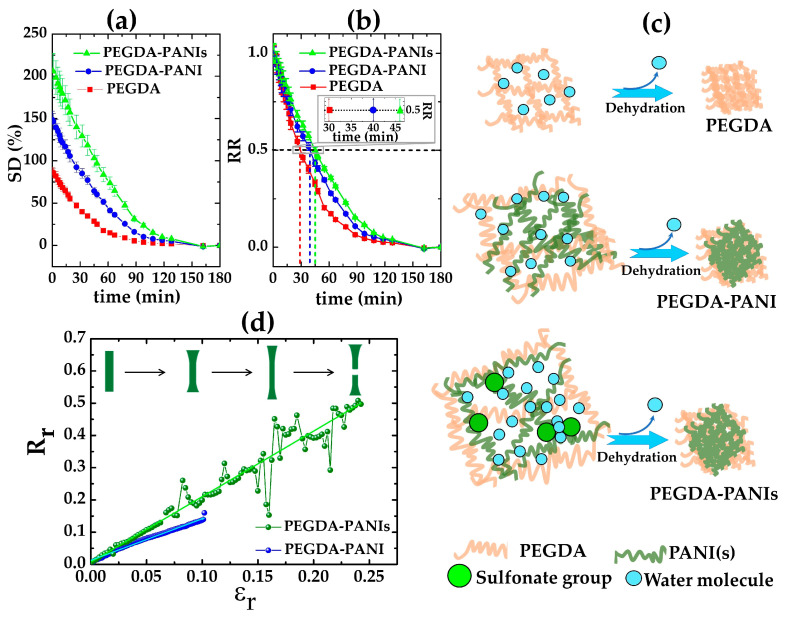
Trend of (**a**) *SD* and (**b**) *RR* values derived at various drying-time intervals for PEGDA–PANIs, PEGDA–PANI, and PEGDA samples; (**c**) Representation of the possible mechanism of dehydration and shrinking; (**d**) Trend of the relative resistance ($R_{r}$) increases as the relative elongation ($\varepsilon_{r}$) for PEGDA–PANIs and PEGDA–PANI samples.

In order to verify if the water affinity and permeability of the PEGDA hydrogel matrix are altered by the presence of the CPs filler, the swelling capacity was measured for both PEGDA–PANIs and PEGDA–PANI samples. In Figure 3a–c, the trend of the swelling degree (*SD*) and the water retention ratio (*RR*) derived at various drying time intervals, along with a representation of the possible dehydration and shrinking mechanism for composites and PEGDA matrix, are reported.

*SD* values of about 210, 150, and 85% were calculated for PEGDA–PANIs, PEGDA–PANI, and PEGDA samples, respectively. This means that the PANI(s) fillers allow for incorporating a greater water content [2,3,81,82]. Notably, PEGDA–PANIs approach the shrunk configuration (i.e., the *SD*(%) = 0) later than both PEGDA–PANI and PEGDA samples (Figure 3a). As shown in Figure 3b, it is possible to note that the water *RR* values of PEGDA–PANIs, PEGDA–PANI, and reference PEGDA halve after about 45, 40, and 30 min, respectively. Once more, this result corroborates that the water retention capacity of the reference PEGDA substrate is lower than that of the composite hydrogels. On the contrary, PEGDA–PANIs display a water-retaining behavior with the slowest kinetics for dehydration and shrinking processes. As schematically depicted in Figure 3c, this outcome can be plausibly explained by the different chemical compositions of the samples. The PEGDA chains are known for their ability to absorb and retain a remarkable content of water molecules through hydrogen-bonding networks [7,9]. Such a property, combined with the presence of hydrophilic groups on the PANI filler, reasonably contributes to the capability of PEGDA–PANI to retain water better than the reference PEGDA hydrogel [2,3]. In turn, the synergistic contribution of these factors with the additional interactions between the water molecules and the sulfonate moieties on the PANIs backbones are expected to further enhance the uptake sites and *RR* for PEGDA–PANIs samples.

The electrical conductivity (σ) values measured by the four-probe technique in the investigated range of potential are about 1.5 × 10^−3^ S/m and 2.5 × 10^−2^ S/m for PEGDA–PANIs and PEGDA–PANI hydrogels, respectively. Due to the insulating nature of PEGDA materials, this result points out that the in situ syntheses of PANIs and PANI phases effectively allow for the production of 3D electrically conductive pathways inside the PEGDA-insulating matrix.

To investigate the electromechanical properties of the produced flexible and electrically conductive PEGDA–PANIs and PEGDA–PANI hydrogels, the change of electrical resistance as a function of the elongation is reported for both the samples in Figure 3d. An increase of relative resistance ($R_{r}$) versus the relative elongation ($\varepsilon_{r}$) is observed as a general trend for both composites. This result can be explained by considering that the elongation of an electrically conductive and stretchable object typically induces significant deformations in its size and geometry. According to Ohm’s second law ($R=\rho \frac{l}{s}$, where *ρ* is the resistivity of the object), the respective increasing and reduction of length (*l*) and cross-section (*s*) (Figure 3d) produces a gradual increasing of the electrical resistance *R* as a function of the elongation degree. In addition, it should be considered that the electrical conductivity of PEGDA–PANI(s) hydrogels is due to the formation of percolation networks among PANI(s) chains homogeneously and finely grown within the PEGDA matrix. In this view, the stretching operation reasonably modifies the entanglement of the PANI(s) chains, lowering the number of connections among the CP units. This occurrence hampers the charge carriers’ transport along PANI(s) units, reducing the interchain charge hopping with the increasing of the elongation values. Under these circumstances, the synergic contribution of the deformation processes and the reduction of the percolative networks gradually increases the hydrogels’ electrical resistance R until the breakpoint. In this context, it is worth noting that elongation at the breakpoint is more than double for the PEGDA–PANIs (~25%) than for the PEGDA–PANI (~10%) system. In line with the swelling degree analyses, this behavior is plausibly related with different natures of the produced hydrogels. Considering that the electromechanical measurements are performed at a 40% humidity in the air, the sulphonic groups on PANIs chains reasonably facilitate the incorporation of water molecules from the environment. As shown in Figure 3c, this enriched chemical composition favors the interchain polymer interactions, producing a PEGDA–PANIs hydrogel that is softer and less brittle than the PEGDA–PANI hydrogel. In the light of these results, the proposed in situ synthesis approaches effectively allow for the production of ECHs that successfully combine properties of water retention, electrical conductivity, and flexibility.

#### 2.2.3. Electrochemical Activity

**Figure 4:**
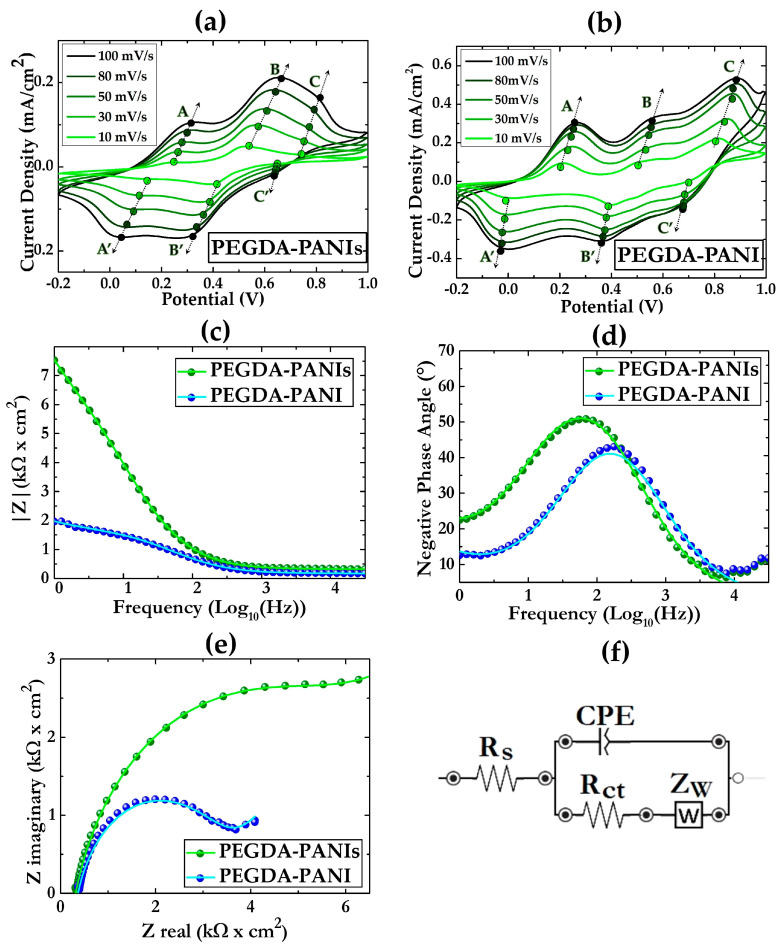
CV curves at 10, 30, 50, 80, and 100 mV/s for (**a**) PEGDA–PANIs and (**b**) PEGDA–PANI in 1 M HCl solution. Experimental (dots) and fitted (straight line) (**c**) Bode, (**d**) Negative phase angle, and (**e**) Nyquist EIS curves recorded for PEGDA–PANIs and PEGDA–PANI in 1 M HCl solution; (**f**) Equivalent electrical circuit used to fit experimental data, where: R_s_ is the solution resistance, CPE is the constant phase element related to double layer capacitance at the electrode surface, R_ct_ is the charge transfer resistance, and Z_w_ is the Warburg resistance.

The CV response and EIS behavior of the produced ECHs were tested in a 1 M HCl aqueous solution (Figure 4).

Analogously to what was found for PANIs and PANI (Figure 1), the CV curves of PEGDA–PANIs and PEGDA–PANI (Figure 4a,b) show rather similar profiles, featured by three pairs of quasi-reversible peaks such as AA’, BB’, and CC’. However, the CV peaks of PEGDA–PANIs and PEGDA–PANI are less sharp with respect to those of the respective PANIs and PANI fillers. This result is reasonably explained by considering that the host PEGDA matrix can hamper the conformational changes of PANI chains from benzenoid to quinonoid form and vice versa. Comparing the two composite CV profiles (Figure 4a,b), PEGDA–PANI shows sharper and more intense peaks in respect to PEGDA–PANIs samples. This loose electrochemical activity is plausibly due to the increasing steric interactions due to the sulfonic functionalities along the PANIs backbone. However, it is to be noted that for both ECHs, the peak currents increase as a function of the scan rate, indicating that the settled methodology effectively enables the production of systems exploitable for electrochemical devices’ assembling.

In order to provide greater feedback for these considerations, EIS measurements were performed on both ECHs. The experimental data were fitted by a standard Randles cell (RC) equivalent circuit model by means of ZView software. In Figure 4c–e the EIS curves recorded for PEGDA–PANIs and PEGDA–PANI samples in 1 M HCl solution are reported. As shown from the diagrams, the EIS plots show minimal differences in the profiles of both hydrogels. Yet, the impedance values collected for PEGDA–PANIs are higher than those of PEGDA–PANI. This is a result that can be explained once again by the presence of sulfonic groups on the PANIs chains, which affect the quality of the charge transport of the ECH filler. Nevertheless, the behavior of both hydrogels can be fitted by the same equivalent circuit, which is a simplified version of the Randles equivalent circuit (Figure 4f). Specifically, the circuit elements are the solution resistance (R_s_), the constant phase element related to double-layer capacitance at the electrode surface (CPE), the charge transfer resistance (R_ct_), and the Warburg resistance (Z_w_). Qualitative information on the magnitude of these elements is reported in Table S1. Despite minimal variations in a few parameters, such as R_s_ and CPE, that can presumably suggest a little contribution of the samples’ surface morphology to the complex impedance [75,83], we can observe a significant difference for the R_ct_ and Z_w_ values. This can be due to the steric hindrance of the sulfonic functionalities, which are expected to hamper the ion exchange processes occurring between the PEGDA–PANIs surfaces and the surrounding electrolyte solution. 

All these outcomes suggest that the resistive interface phenomena are greater for PEGDA–PANIs than PEGDA–PANI. However, it is fundamental to note that the absolute impedance values at 1 Hz are lower than 8 kΩ for both the investigated ECHs, as well as that these values are in line with those recorded for other PANI-based composites commonly proposed as electrode materials for sensing applications [52,53,54,55,56,57].

#### 2.2.4. CVs as a Function of pH

The good electrical and electrochemical properties, the great flexibility, and the intrinsic biocompatibility of PEGDA–PANI materials [2,23] suggested to test the 3D printed ECHs as free-standing patch electrodes for pH monitoring.

**Figure 5:**
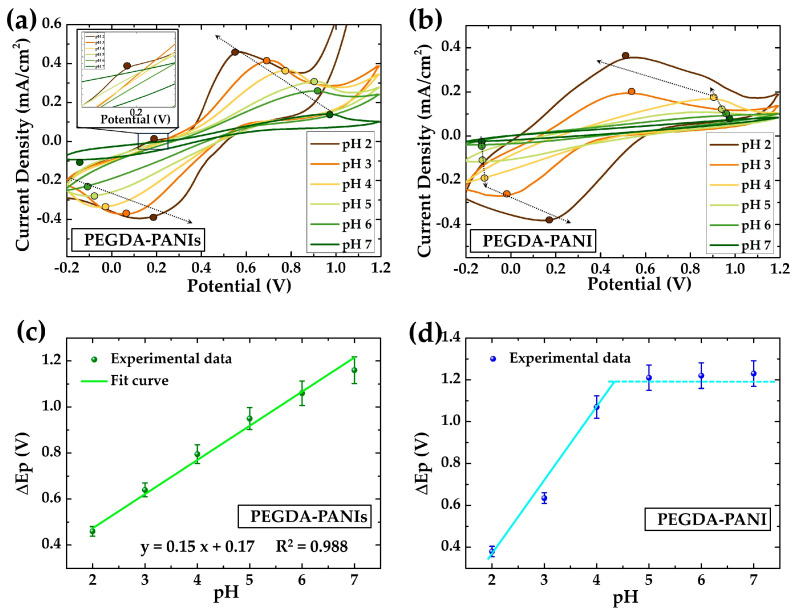
CV curves recorded at the scan rate of 50 mV/s in 0.1 M NaCl solution at different pHs by (**a**) PEGDA–PANIs and (**b**) PEGDA–PANI; trend of the difference between the anodic and cathodic peaks’ position versus pH for (**c**) PEGDA–PANIs (green line: fit curve) and (**d**) PEGDA–PANI (cyan line: eyes guideline).

The pH-responsive properties were studied between pH 2.0 and 7.0, which is the clinical range of variation of most biofluids such as sweat, vaginal fluid, gastroesophageal reflux and urine [58,59,60,61]. The pH sensing tests were conducted on PEGDA–PANIs and PEGDA–PANI parallelepiped samples (Figure 2a,b). The voltammograms, shown in Figure 5a,b, were recorded at the scan rate of 50 mV s^−1^ within the potential range between −0.2 V and +1.2 V in 0.1 M NaCl solutions properly buffered at pH 2, 3, 4, 5, 6, and 7 to purposefully observe the redox peaks of PANI and PANIs fillers. In Figure 5c,d, the linear fittings of the difference between the oxidation and reduction peaks’ positions are plotted versus the pH of the tested solutions.

The CV curves acquired for both ECHs (Figure 5a,b) show a single redox peak couple reasonably due to the electrochemistry of the PANIs and PANI fillers. In particular, the CV curve recorded at pH 2 for PEGDA–PANIs exhibits an additional oxidation peak at around +0.2 V, plausibly obtained by hydrogen adsorption/desorption processes with PANIs phase. These phenomena are obviously favored by the faster charge transfer activity and the more intense electrochemical response under strong acidic conditions. Interestingly, PEGDA–PANIs show a greater electrochemical activity in all tested solutions, in full agreement with what was found for the only fillers (Figure 1). Specifically, comparing the activity of the two ECHs at neutral pH (0.1 M NaCl solution), the marked improvement due to the PANIs component is evidenced by the persistence of redox reactions. In fact, PEGDA–PANIs material ensures a sustained electrochemical activity up to pH 7, whilst PEGDA–PANI produces low current densities approaching a neutral environment. As previously discussed, this different behavior can be related to the presence of the sulfonic groups along the polymeric chains of PANIs, which act as self-doping agents. 

By analyzing the CV curves in more detail, we can see how the redox peaks spread apart as the pH increases for both ECHs. However, while this trend is appreciable for the entire pH range for PEGDA–PANIs (Figure 5a), it is only notable between pH 2 and 4 for PEGDA–PANI (Figure 5b). These observations are summarized in the diagram showing the separation between the anodic and cathodic peaks ΔE_p_ as a function of pH (Figure 5c,d). For PEGDA–PANI, we can find a noise regime between pH 7 and 4, after which the hydrogel abruptly increases its response under acidic conditions (Figure 5d). Conversely, PEGDA–PANIs show a remarkably good linear response in the same dynamic range (Figure 5c). The equation of the linear plot and the correlation coefficient (R^2^) values are provided in the figure. In particular, the slope of the linear fit provides a sensitivity of pH measurement of 0.15 ΔE_p_/pH. These results convincingly underline the superior sensing efficiency of PEGDA–PANIs with respect to that of PEGDA–PANI. Again, this feature can be explained with the chemical structure of the interacting PEGDA and PANIs chains (Figure 3c). Specifically, the sulfonic groups on the PANIs backbone, which act as inner dopant anions, can interact with the protonated water molecules entrapped within the meshes of the PEGDA matrix by providing a general enhanced electrochemical response. In this perspective, PEGDA–PANIs hydrogel proves to be a potentially ideal material for electrochemical applications in an extended pH range. The electrochemical response of PEGDA–PANI and PEGDA–PANIs is in line with that of other PANI-based composite materials (Table S2) [81,84]. In addition, the great flexibility, *SD,* and water uptake capability are added values that make the produced ECHs excellent candidates for the realization of electrical devices for biomedical electronic applications.

## 3. Conclusions

The idea behind this work was to develop a methodology for the production by stereolithography of a self-standing hydrogel system that had ideal electrical, electrochemical, and flexibility properties for the realization of a wearable sensor capable of pH monitoring under physiological conditions. The novelty here proposed lies in exploiting the in situ synthesis of PANIs conducting polymer within the PEGDA matrix to prepare electroconductive composite hydrogels with improved electro–chemo–mechanical, swelling degree, and water-retention capacity without post-processing treatments. 

SEM, IR, Raman spectroscopy, XRD, I-V, and CV analyses were performed to assess the co-polymerization reaction between ANI and ANIs monomers in terms of morphology, structure, electrical, and electrochemical properties of the produced PANIs system. It was found that the presence of the sulfonic groups makes CPs able to support redox reactions within the 2–7 pH range. This feature was properly carried over to the PEGDA–PANIs composite, which has been shown to possess superior water absorption/retention properties, good electrical conductivity, and extensive electrochemistry over a wide pH range.

As a proof of concept, the PEGDA–PANIs electrically conductive hydrogel was successfully tested as electrode material for a pH-monitoring device working in the physiological range between 2 and 7, providing a linear detection response in the whole investigated range.

In the view of the overall collected results, the 3D printability of the PEGDA inks with well-defined and complex shapes, coupled with the biocompatibility, flexibility, water retention, and the tunability of the electrical and electrochemical properties of the produced PEGDA–PANIs composites, paves the way for developing hydrogels not only for pH monitoring across the clinically relevant range of most biofluids’ variation but also potentially exploitable for a series of new emerging fields. In addition, thanks to the 3D printing technology it becomes possibility to customize each flexible sensor for an easy fitting of any specific application. Thus, through the collaboration of researchers in different fields such as chemistry, materials science, medicine, and engineering, it will be possible to develop flexible and customizable devices from smart biomedical sensors to new artificial tissues.

## 4. Materials and Methods

### 4.1. Chemicals

Aniline (ANI), aniline-2-sulfonic acid (ANIs), ammonium persulfate (APS), ferric chloride (FeCl_3_), sodium chloride (NaCl), hydrochloric acid (HCl), dimethyl sulfoxide (DMSO), acetone, bis(2,4,6-trimethylbenzoyl)-phenylphosphineoxide (Irgacure 819), and poly-ethylene glycol diacrylate (PEGDA) with MW ∼575 Da were purchased by Merk. 

### 4.2. 3D Printing of PEGDA Substrates

**Figure 6:**
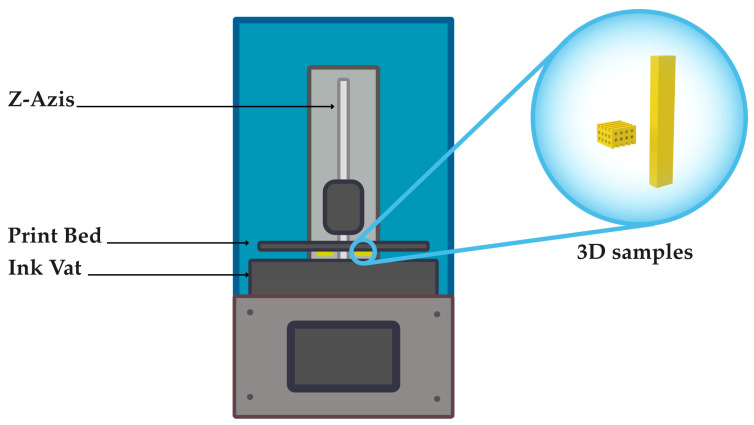
Scheme of the SLA printer apparatus along with the CAD models of the 3D-printed PEGDA samples with woodpile and parallelepiped geometries.

1% *w*/*w* Irgacure 819 was dissolved in a 20% *w*/*w* PEGDA/acetone solution to obtain a printable ink. PEGDA substrates were printed by means of a commercial ELEGOO Mars MSLA printer equipped with a 405-nm light lamp and characterized by a printing resolution of 47 µm on the XY plane and 1.25 µm on the Z axis. PEGDA objects with different geometries were designed with a 3D Computer-Aiding Design (CAD) software and converted into stereolithography (STL) files, which were successively transcribed into printer-readable files by Chitubox software. The optimized thickness of each layer of samples was 50 µm and the UV light exposure was 60 s. After printing, each PEGDA sample was rinsed with acetone to remove the ink excess, further exposed to UV light for 10 min, and then left to dry at room temperature. In Figure 6, the scheme of the SLA printer apparatus, along with the CAD models of the 3D-printed PEGDA samples, with different geometries are reported. A picture of the ELEGOO Mars MSLA printer apparatus is shown in Figure S2.

### 4.3. Preparation of PANI-Based Reference Samples

Polyaniline-based copolymer sample was obtained starting from 1 M/1M ANI/ANIs solution DMSO. For the sake of simplicity, the sample obtained with this combination of precursors will be identified with the label PANIs from here on. For the preparation of PANI sample, a solution of 2 M ANI in DMSO was used. A 0.05 M APS in a 1 M HCl solution was used as initiator for all monomer polymerizations. To rule out the possible contribution of APS molecules in introducing sulfonate groups on the CP backbone, PANI and PANIs polymers were additionally prepared by replacing the APS with 0.1 M FeCl_3_ as oxidant agent (experimental conditions in Table S3). Once the polymerizations were complete, the samples were repeatedly washed with ethanol and distilled water to reach a pH ≈ 6 and finally collected in form of powders. For some analysis, the samples were also compelled in pellet form.

### 4.4. Preparation of PEGDA–PANIs Composites

To produce PEGDA–PANIs composites, PEGDA samples were soaked for 24 h in a 1 M/1 M ANI/ANIs solution in DMSO. For preparation of PEGDA–PANI samples, a soaking pretreatment in pure ANI for 24 h was necessary to guarantee the permeation of aniline molecules within the PEGDA matrix substrates. After that, the ANI-impregnated PEGDA substrates were transferred in 2 M ANI solution in DMSO. For preparation of both composite samples, a 0.05 M APS in a 1 M HCl solution was used as radical initiator for chemical oxidative polymerization. Specifically, the APS oxidizing solution was added at a 1 mL/min flow rate to the ANI/ANIs and ANI solutions containing PEGDA substrates kept at 0–5 °C by means of an ice bath. Under these conditions, an equimolar ratio between precursors and oxidant was achieved for each polymerization reaction. All the syntheses lasted for 24 h, and the products were filtered and repetitively washed by water and ethanol. In Table S4 the samples’ names and details on the synthesis conditions are listed. To verify the reproducibility of the process, three replicas of each sample were prepared and analyzed.

### 4.5. Characterization Techniques

The morphology of samples was analyzed by means of a Zeiss Leo Supra 35 field emission scanning electron microscope (FEG–SEM) equipped with a high-resolution secondary electron detector operating at 10 keV. Sample characterization was performed using secondary electrons with a 3-kV accelerating voltage and a 20-μm aperture size.

The molecular structure of samples was studied by Raman and FT–IR spectroscopy. Raman spectra were collected by an XploRA ONE™ Raman Microscope (Horiba) under a 785-nm laser excitation, a 1% laser power, and a diffraction grating of 2400 gr/mm. Raman spectra were normalized and deconvolved by using Lorentz function lines to derive the parameters of position, amplitude, and integrated intensity for each peak. FT–IR spectra were acquired by a Madison WI-Is 50 Thermo Scientific Inc. spectrophotometer, which is equipped with a diamond cell at single reflection for measurements in attenuated total reflectance (ATR). Spectra were collected in the 4000 and 500 cm^−1^ spectral region.

The crystalline structure of samples was investigated by XRD. XRD patterns were recorded using a PW1730 Philips diffractometer in a Bragg Brentano configuration, equipped with a copper tube producing Cu K_α_ radiation of 1.5406 Å wavelength. The X-ray diffractograms were acquired for 6 h in the 2θ° range between 5–60° at 0.01° step. The XRD patterns were deconvolved to Voigt function lines to derive the X-ray Peak Profile Analysis (XPPA).

Electrical conductibility of samples was evaluated by I–V characteristics collected by using the four-probe technique. The probes were connected to a Keithley 6221 current source and a Keithley 2700 multimeter, controlled by LabVIEW interface. The electrical conductivity of the samples was calculated according to the procedure reported in [63]. 

Electrochemical properties of samples were studied by cyclic voltammetry (CV) and electrochemical impedance spectroscopy (EIS). CV measurements were performed in a standard one-compartment three-electrode cell by means of a Palm Sense compact electrochemical workstation. For PANIs and PANI samples, the working electrode was prepared by depositing PANI and PANIs phases on platinum foils. For PEGDA–PANIs and PEGDA-PANI samples, the working electrode was assembled by providing a platinum contact to the backside of the 3D objects. For all the electrochemical measurements, a saturated calomel electrode (SCE) and a Pt foil were used as the reference and counter electrode, respectively. CV curves were conducted in the −0.2 V ÷ +1.0 V potential range at scan rates of 10–30–50–80–100 mV/s in 1 M HCl solution. To analyze the pH effect on samples’ electroactivity, CV tests were performed in the same potential range and at 50 mV/s scan rate in 0.1 M NaCl solutions buffered at pH ranging from 2 to 7. EIS measurements were conducted in 1 M HCl solution at frequencies from 1 Hz to 40 kHz by applying a sinusoidal wave of 50 mV of amplitude superimposed to a voltage bias of 0 V. Fitting operation and data analysis were performed by using PSTrace software.

The swelling degree of samples was measured by developing the following protocol: (i) The dried hydrogel samples were immersed in distilled water for 2 h at 30 °C; (ii) The samples were quickly removed, wiped dry to remove excess surface water, and finally weighed to determine the wet mass (Mw); (iii) The samples’ dried weight (Md) was determined after dry at 30 °C for 4 h. The swelling degree (*SD*) and the water retention ratio (*RR*) at 30 °C were derived at different drying times according to the following Equations (3) and (4) [13], respectively:

(3) $$SD=\frac{M_{t}-M_{d}}{M_{d}}$$

(4) $$RR=\frac{M_{t}-M_{d}}{M_{s}-M_{d}}$$

where $M_{d}$, $M_{s}$ and $M_{t}$ are the weight of the dried hydrogel, the swollen hydrogel at the time point *t* = 0, and the sample at the testing time (*t*), respectively. 

Electromechanical properties were evaluated by means of a customized apparatus to measure the change in electrical resistance as a function of the elongation. While measuring the voltage through a 10-mm parallelepiped sample by the application of a fixed current of 300 nA (Keithley 6221), the sample was elongated at a fixed speed of 600 µm/min by using a motorized linear stage interfaced with a LabVIEW software. The variance of relative resistance and elongation were calculated as described in [85]. Characterization analyses were performed at least three times to verify reproducibility of prepared sample response.

## Data Availability

The data that support the findings of this study are available from the corresponding author upon reasonable request.

## Supplementary Materials

The following supporting information can be downloaded at: https://www.mdpi.com/article/10.3390/gels9100784/s1. Note S1: Raman spectroscopy analysis; Figure S1: The IR spectra of PANI and PANIs; Note S2: XRD analysis; Table S1: EIS parameters for PEGDA-PANIs and PEGDA-PANI hydrogels; Table S2: Comparison of PANI sensors in terms of sensitivity, pH range analyzed and preparation methods; Figure S2: Picture of the ELEGOO Mars MSLA printer apparatus; Table S3: List of the experimental conditions for ANIs and ANI polymerization processes; Table S4: List of experimental conditions for the in-situ polymerization processes.

## References

[1] Kougkolos G., Golzio M., Laudebat L., Valdez-Nava Z., Flahaut E. (2023). Hydrogels with Electrically Conductive Nanomaterials for Biomedical Applications. J. Mater. Chem. B.

[2] Guarino V., Alvarez-Perez M.A., Borriello A., Napolitano T., Ambrosio L. (2013). Conductive PANi/PEGDA Macroporous Hydrogels For Nerve Regeneration. Adv. Healthc. Mater..

[3] Wu Y., Chen Y.X., Yan J., Yang S., Dong P., Soman P. (2015). Fabrication of Conductive Polyaniline Hydrogel Using Porogen Leaching and Projection Microstereolithography. J. Mater. Chem. B.

[4] Heo D.N., Lee S.J., Timsina R., Qiu X., Castro N.J., Zhang L.G. (2019). Development of 3D Printable Conductive Hydrogel with Crystallized PEDOT:PSS for Neural Tissue Engineering. Mater. Sci. Eng. C.

[5] Rogers Z.J., Zeevi M.P., Koppes R., Bencherif S.A. (2020). Electroconductive Hydrogels for Tissue Engineering: Current Status and Future Perspectives. Bioelectricity.

[6] Guo B., Zhong Y., Chen X., Yu S., Bai J. (2023). 3D Printing of Electrically Conductive and Degradable Hydrogel for Epidermal Strain Sensor. Compos. Commun..

[7] Athukorala S.S., Tran T.S., Balu R., Truong V.K., Chapman J., Dutta N.K., Roy Choudhury N. (2021). 3D Printable Electrically Conductive Hydrogel Scaffolds for Biomedical Applications: A Review. Polymers.

[8] Aggas J.R., Abasi S., Phipps J.F., Podstawczyk D.A., Guiseppi-Elie A. (2020). Microfabricated and 3-D Printed Electroconductive Hydrogels of PEDOT:PSS and Their Application in Bioelectronics. Biosens. Bioelectron..

[9] Distler T., Boccaccini A.R. (2020). 3D Printing of Electrically Conductive Hydrogels for Tissue Engineering and Biosensors—A Review. Acta Biomater..

[10] Zhang C., Li Y., Kang W., Liu X., Wang Q. (2021). Current Advances and Future Perspectives of Additive Manufacturing for Functional Polymeric Materials and Devices. SusMat.

[11] Lee J.Y., An J., Chua C.K. (2017). Fundamentals and Applications of 3D Printing for Novel Materials. Appl. Mater. Today.

[12] Turner B.N., Strong R., Gold S.A. (2014). A Review of Melt Extrusion Additive Manufacturing Processes: I. Process Design and Modeling. Rapid Prototyp. J..

[13] Ul Haq A., Montaina L., Pescosolido F., Carotenuto F., Trovalusci F., De Matteis F., Tamburri E., Di Nardo P. (2023). Electrically Conductive Scaffolds Mimicking the Hierarchical Structure of Cardiac Myofibers. Sci. Rep..

[14] Politi S., Tamburri E., Carcione R., Lavecchia T., Angjellari M., Terranova M.L. Innovative Preparation Processes and Structural Characteristics of 3D Printable Polymer-Based Nanocomposites. Proceedings of the AIP Conference Proceedings.

[15] Wallin T.J., Pikul J., Shepherd R.F. (2018). 3D Printing of Soft Robotic Systems. Nat. Rev. Mater..

[16] Zhang Y.F., Zhang N., Hingorani H., Ding N., Wang D., Yuan C., Zhang B., Gu G., Ge Q. (2019). Fast-Response, Stiffness-Tunable Soft Actuator by Hybrid Multimaterial 3D Printing. Adv. Funct. Mater..

[17] Truby R.L., Wehner M., Grosskopf A.K., Vogt D.M., Uzel S.G.M., Wood R.J., Lewis J.A. (2018). Soft Somatosensitive Actuators via Embedded 3D Printing. Adv. Mater..

[18] Foster C.W., Elbardisy H.M., Down M.P., Keefe E.M., Smith G.C., Banks C.E. (2020). Additively Manufactured Graphitic Electrochemical Sensing Platforms. Chem. Eng. J..

[19] Rim Y.S., Bae S.H., Chen H., De Marco N., Yang Y. (2016). Recent Progress in Materials and Devices toward Printable and Flexible Sensors. Adv. Mater..

[20] Murr L.E. (2016). Frontiers of 3D Printing/Additive Manufacturing: From Human Organs to Aircraft Fabrication. J. Mater. Sci. Technol..

[21] Gisario A., Kazarian M., Martina F., Mehrpouya M. (2019). Metal Additive Manufacturing in the Commercial Aviation Industry: A Review. J. Manuf. Syst..

[22] Angjellari M., Tamburri E., Montaina L., Natali M., Passeri D., Rossi M., Terranova M.L. (2017). Beyond the Concepts of Nanocomposite and 3D Printing: PVA and Nanodiamonds for Layer-by-Layer Additive Manufacturing. Mater. Des..

[23] Montaina L., Carcione R., Pescosolido F., Montalto M., Battistoni S., Tamburri E. (2022). Three-Dimensional-Printed Polyethylene Glycol Diacrylate-Polyaniline Composites by in Situ Aniline Photopolymerization: An Innovative Biomaterial for Electrocardiogram Monitoring Systems. ACS Appl. Electron. Mater..

[24] Smith C.F., Tollemache N., Covill D., Johnston M. (2018). Take Away Body Parts! An Investigation into the Use of 3D-Printed Anatomical Models in Undergraduate Anatomy Education. Anat. Sci. Educ..

[25] Zarek M., Layani M., Cooperstein I., Sachyani E., Cohn D., Magdassi S. (2016). 3D Printing of Shape Memory Polymers for Flexible Electronic Devices. Adv. Mater..

[26] Liang K., Carmone S., Brambilla D., Leroux J.C. (2018). 3D Printing of a Wearable Personalized Oral Delivery Device: A First-in-Human Study. Sci. Adv..

[27] Ma X., Liu J., Zhu W., Tang M., Lawrence N., Yu C., Gou M., Chen S. (2018). 3D Bioprinting of Functional Tissue Models for Personalized Drug Screening and in Vitro Disease Modeling. Adv. Drug Deliv. Rev..

[28] Bose S., Ke D., Sahasrabudhe H., Bandyopadhyay A. (2018). Additive Manufacturing of Biomaterials. Prog. Mater. Sci..

[29] Valentine A.D., Busbee T.A., Boley J.W., Raney J.R., Chortos A., Kotikian A., Berrigan J.D., Durstock M.F., Lewis J.A. (2017). Hybrid 3D Printing of Soft Electronics. Adv. Mater..

[30] Gibson I., Rosen D., Stucker B. (2015). Additive Manufacturing Technologies: 3D Printing, Rapid Prototyping, and Direct Digital Manufacturing.

[31] Gonzalez G., Roppolo I., Pirri C.F., Chiappone A. (2022). Current and Emerging Trends in Polymeric 3D Printed Microfluidic Devices. Addit. Manuf..

[32] Isreb A., Baj K., Wojsz M., Isreb M., Peak M., Alhnan M.A. (2019). 3D Printed Oral Theophylline Doses with Innovative ‘Radiator-like’ Design: Impact of Polyethylene Oxide (PEO) Molecular Weight. Int. J. Pharm..

[33] Scordo G., Bertana V., Ballesio A., Carcione R., Marasso S.L., Cocuzza M., Pirri C.F., Manachino M., Gomez M.G., Vitale A. (2021). Effect of Volatile Organic Compounds Adsorption on 3D-Printed Pegda:Pedot for Long-Term Monitoring Devices. Nanomaterials.

[34] Battistoni S., Cocuzza M., Marasso S.L., Verna A., Erokhin V. (2021). The Role of the Internal Capacitance in Organic Memristive Device for Neuromorphic and Sensing Applications. Adv. Electron. Mater..

[35] Poddar A.K., Patel S.S., Patel H.D. (2021). Synthesis, Characterization and Applications of Conductive Polymers: A Brief Review. Polym. Adv. Technol..

[36] Kar P. (2013). Doping in Conjugated Polymers.

[37] Passeri D., Biagioni A., Rossi M., Tamburri E., Terranova M.L. (2013). Characterization of Polyaniline-Detonation Nanodiamond Nanocomposite Fibers by Atomic Force Microscopy Based Techniques. Eur. Polym. J..

[38] Passeri D., Tamburri E., Terranova M.L., Rossi M. (2015). Polyaniline–Nanodiamond Fibers Resulting from the Self-Assembly of Nano-Fibrils: A Nanomechanical Study. Nanoscale.

[39] Doan T.C.D., Ramaneti R., Baggerman J., Van Der Bent J.F., Marcelis A.T.M., Tong H.D., Van Rijn C.J.M. (2012). Carbon Dioxide Sensing with Sulfonated Polyaniline. Sens. Actuators B Chem..

[40] Yue J., Wang Z.H., Cromack K.R., Epstein A.J., MacDiarmid A.G. (1991). Effect of Sulfonic Acid Group on Polyaniline Backbone. J. Am. Chem. Soc..

[41] Wei X.L., Wang Y.Z., Long S.M., Bobeczko C., Epstein A.J. (1996). Synthesis and Physical Properties of Highly Sulfonated Polyaniline. J. Am. Chem. Soc..

[42] Wei X., Epstein A.J. (1995). Synthesis of Highly Sulfonated Polyaniline. Synth. Met..

[43] Yue J., Epstein A.J. (1990). Synthesis of Self-Doped Conducting Polyaniline. J. Am. Chem. Soc..

[44] Yang Y., Min Y., Wu J.C., Hansford D.J., Feinberg S.E., Epstein A.J. (2011). Synthesis and Characterization of Cytocompatible Sulfonated Polyanilines. Macromol. Rapid Commun..

[45] Bernard M.C., Hugot-Le Goff A. (2006). Quantitative Characterization of Polyaniline Films Using Raman Spectroscopy: II. Effects of Self-Doping in Sulfonated Polyaniline. Electrochim. Acta.

[46] Yue J., Gordon G., Epstein A.J. (1992). Comparison of Different Synthetic Routes for Sulphonation of Polyaniline. Polymer.

[47] Brochocka A., Nowak A., Zajączkowska H., Sieradzka M. (2021). Chemosensitive Thin Films Active to Ammonia Vapours. Sensors.

[48] Medi B., Bahramian A., Nazari V. (2021). Synthesis and Characterization of Conducting Polyaniline Nanostructured Thin Films for Solar Cell Applications. JOM.

[49] Akhlaq M., Khan Z.S., Mohamed A., Ibrahim M., Fouly A., Mohamed A., Fathelbab A.M.R., Akber H.J., Ibrahim I.M., Razeg K.H. (2020). Hydrothermal Synthesis of Polyaniline Nano-Fibers as H_2_S Gas Sensor You May Also like Synthesis and Characterization of Electro-Spun TiO_2_ and TiO_2_-SnO_2_ Composite Nano-Fibers for Application in Advance Generation Solar Cells Enhancing the Tribological Performance of Epoxy Composites Utilizing Carbon Nano Fibers Additives for Journal Bearings Hydrothermal Synthesis of Polyaniline Nano-Fibers as H 2 S Gas Sensor. J. Phys. Conf. Ser..

[50] Battistoni S., Verna A., Marasso S.L., Cocuzza M., Erokhin V. (2020). On the Interpretation of Hysteresis Loop for Electronic and Ionic Currents in Organic Memristive Devices. Phys. Status Solidi.

[51] Battistoni S., Erokhin V., Iannotta S. (2017). Emulation with Organic Memristive Devices of Impairment of LTP Mechanism in Neurodegenerative Disease Pathology. Neural Plast..

[52] Kadri Y., Bekri-Abbess I., Herrasti P. (2022). Highly Sensitive Enzyme-Free Sensor Based on a Carbon Paste Electrode Modified with Binary Zinc Oxide/Polyaniline Nanocomposites for Dopamine, Ascorbic Acid and Uric Acid Sensing. Electroanalysis.

[53] Chi M., Zhu Y., Jing L., Wang C., Lu X. (2019). Fabrication of Oxidase-like Polyaniline-MnO2 Hybrid Nanowires and Their Sensitive Colorimetric Detection of Sulfite and Ascorbic Acid. Talanta.

[54] Aryal K.P., Jeong H.K. (2021). Simultaneous Determination of Ascorbic Acid, Dopamine, and Uric Acid with Polyaniline/Hemin/Reduced Graphite Oxide Composite. Chem. Phys. Lett..

[55] Shen Y., Zheng L. (2023). Polyaniline-Poly (Methylene Blue) Nano-Rod Composites as an Electrochemical Sensor for Sensitive Determination of Ascorbic Acid. Int. J. Electrochem. Sci..

[56] Naghib S.M., Behzad F., Rahmanian M., Zare Y., Rhee K.Y. (2020). A Highly Sensitive Biosensor Based on Methacrylated Graphene Oxide-Grafted Polyaniline for Ascorbic Acid Determination. Nanotechnol. Rev..

[57] Bilal S., Akbar A., Shah A.U.H.A. (2019). Highly Selective and Reproducible Electrochemical Sensing of Ascorbic Acid Through a Conductive Polymer Coated Electrode. Polymers.

[58] Pal A., Nadiger V.G., Goswami D., Martinez R.V. (2020). Conformal, Waterproof Electronic Decals for Wireless Monitoring of Sweat and Vaginal PH at the Point-of-Care. Biosens. Bioelectron..

[59] Gao W., Nyein H.Y.Y., Shahpar Z., Tai L.C., Wu E., Bariya M., Ota H., Fahad H.M., Chen K., Javey A. (2017). Wearable Sweat Biosensors. Tech. Dig. Int. Electron Devices Meet. IEDM.

[60] Roman S., Gyawali C.P., Savarino E., Yadlapati R., Zerbib F., Wu J., Vela M., Tutuian R., Tatum R., Sifrim D. (2017). Ambulatory Reflux Monitoring for Diagnosis of Gastro-Esophageal Reflux Disease: Update of the Porto Consensus and Recommendations from an International Consensus Group. Neurogastroenterol. Motil..

[61] Rose C., Parker A., Jefferson B., Cartmell E. (2015). The Characterization of Feces and Urine: A Review of the Literature to Inform Advanced Treatment Technology. Crit. Rev. Environ. Sci. Technol..

[62] Qiu S., Chen C., Zheng W., Li W., Zhao H., Wang L. (2017). Long-Term Corrosion Protection of Mild Steel by Epoxy Coating Containing Self-Doped Polyaniline Nanofiber. Synth. Met..

[63] Liao Y., Strong V., Chian W., Wang X., Li X.G., Kaner R.B. (2012). Sulfonated Polyaniline Nanostructures Synthesized via Rapid Initiated Copolymerization with Controllable Morphology, Size, and Electrical Properties. Macromolecules.

[64] Lindfors T., Ivaska A. (2005). Raman Based PH Measurements with Polyaniline. J. Electroanal. Chem..

[65] Tamburri E., Orlanducci S., Guglielmotti V., Reina G., Rossi M., Terranova M.L. (2011). Engineering Detonation Nanodiamond—Polyaniline Composites by Electrochemical Routes: Structural Features and Functional Characterizations. Polymer..

[66] Tamburri E., Guglielmotti V., Orlanducci S., Terranova M.L., Sordi D., Passeri D., Matassa R., Rossi M. (2012). Nanodiamond-Mediated Crystallization in Fibers of PANI Nanocomposites Produced by Template-Free Polymerization: Conductive and Thermal Properties of the Fibrillar Networks. Polymer.

[67] Józefowicz M.E., Epstein A.J., Pouget J.P., Masters J.G., Ray A., Sun Y., Tang X., Macdiarmid A.G. (1991). X-ray Structure of Polyanilines. Synth. Met..

[68] Freund M.S., Deore F. (2007). Self-Doped Conductive Polymers.

[69] Heffner G.W., Dahman S.J., Pearson D.S., Gettinger C.L. (1993). The Effect of Molecular Weight and Crystallinity on the Conductivity of a Conducting Polymer. Polymer.

[70] Fan F., Wang W., Holt A.P., Feng H., Uhrig D., Lu X., Hong T., Wang Y., Kang N.G., Mays J. (2016). Effect of Molecular Weight on the Ion Transport Mechanism in Polymerized Ionic Liquids. Macromolecules.

[71] Du X., Xu Y., Xiong L., Bai Y., Zhu J., Mao S. (2014). Polyaniline with High Crystallinity Degree: Synthesis, Structure, and Electrochemical Properties. J. Appl. Polym. Sci.

[72] Popov A., Brasiunas B., Mikoliunaite L., Bagdziunas G., Ramanavicius A., Ramanaviciene A. (2019). Comparative Study of Polyaniline (PANI), Poly(3,4-Ethylenedioxythiophene) (PEDOT) and PANI-PEDOT Films Electrochemically Deposited on Transparent Indium Thin Oxide Based Electrodes. Polymer.

[73] Chen W.C., Wen T.C., Hu C.C., Gopalan A. (2002). Identification of Inductive Behavior for Polyaniline via Electrochemical Impedance Spectroscopy. Electrochim. Acta.

[74] Kuzmany H., Sariciftci N.S. (1987). In Situ Spectro-Electrochemical Studies of Polyaniline. Synth. Met..

[75] Biabangard F., Nazari H., Arefinia R. (2021). Effect of PH on the Electrochemical Properties of Polyaniline Nanoparticle Suspension in Strongly Acidic Solution: An Experimental and Theoretical Study. J. Solid State Electrochem..

[76] Yan B., Yang J., Li Y., Cao Y. (1991). Electrochemical Adsorption of Hydrogen and Various Ions on Polyaniline Film. Reactions Concerning the First Pair of Cyclic Voltammetric Peaks. Synth. Met..

[77] Jamadade V.S., Dhawale D.S., Lokhande C.D. (2010). Studies on Electrosynthesized Leucoemeraldine, Emeraldine and Pernigraniline Forms of Polyaniline Films and Their Supercapacitive Behavior. Synth. Met..

[78] Li C., Mu S. (2005). The Electrochemical Activity of Sulfonic Acid Ring-Substituted Polyaniline in the Wide PH Range. Synth. Met..

[79] Xue P., Zhang X., Chuah Y.J., Wu Y., Kang Y. (2015). Flexible PEGDA-Based Microneedle Patches with Detachable PVP–CD Arrowheads for Transdermal Drug Delivery. RSC Adv..

[80] Pescosolido F., Montaina L., Carcione R., Politi S., Matassa R., Carotenuto F., Nottola S.A., Di Nardo P., Tamburri E., Pescosolido F. (2023). A New Strong-Acid Free Route to Produce Xanthan Gum-PANI Composite Scaffold Supporting Bioelectricity. Macromol. Biosci..

[81] Mir A., Kumar A., Riaz U. (2022). A Short Review on the Synthesis and Advance Applications of Polyaniline Hydrogels. RSC Adv..

[82] Yin X., Zhang Y., Guo Q., Cai X., Xiao J., Ding Z., Yang J. (2018). Macroporous Double-Network Hydrogel for High-Efficiency Solar Steam Generation under 1 Sun Illumination. ACS Appl. Mater. Interfaces.

[83] Yang S., Zhu S., Hong R. (2020). Graphene Oxide/Polyaniline Nanocomposites Used in Anticorrosive Coatings for Environmental Protection. Coatings.

[84] Pyarasani R.D., Jayaramudu T., John A. (2018). Polyaniline-Based Conducting Hydrogels. J. Mater. Sci..

[85] Cochrane C., Koncar V., Lewandowski M., Dufour C. (2007). Design and Development of a Flexible Strain Sensor for Textile Structures Based on a Conductive Polymer Composite. Sensors.

