# Supplementary material for: Self-Standing 3D-Printed PEGDA–PANIs Electroconductive Hydrogel Composites for pH Monitoring
Source: Gels. 2023 Sep 26;9(10):784. doi: 10.3390/gels9100784 (PMC10606186; doi:10.3390/gels9100784)
Supplement: Supplementary file 1 [file gels-09-00784-s001.zip › gels-2612257-supplementary.pdf]

SUPPLEMENTARY INFORMATION

Self-Standing 3D Printed PEGDA-PANIs Electroconductive Hydrogel Composites for pH Monitoring

Rocco Carcione[1]†, Francesca Pescosolido[2,3]†, Luca Montaina[2], Francesco Toschi[4], Silvia Orlanducci[2], Emanuela Tamburri[2,3*] and Silvia Battistoni[1*]

[1] *Consiglio Nazionale delle Ricerche - Institute of Materials for Electronics and Magnetism (CNR-IMEM) Parco Area delle Scienze 37A, Parma 43124, Italy*
[2] *Dip.to di Scienze e Tecnologie Chimiche & UdR INSTM di Roma - Università degli Studi di Roma "Tor Vergata" - Via della Ricerca Scientifica Rome 00133, Italy*
[3] *Centro di Ricerca Interdipartimentale di Medicina Rigenerativa (CIMER), Università degli Studi di Roma "Tor Vergata", Via Montpellier 1, Rome 00133, Italy*
[4] *Istituto di Struttura della Materia - CNR (ISM-CNR), EuroFEL Support Laboratory (EFSL), Monterotondo Scalo 00015, Italy*
* Correspondence: silvia.battistoni@imem.cnr.it, emanuela.tamburri@uniroma2.it
† These authors contributed equally to this work

Note S1: Raman spectroscopy analysis

The single peak around 1170 is related to the C-H bending of the semi-quinoid rings [1,2] while the signals at about 1615 cm^{-1} and 1590 cm^{-1} corresponds to C-C stretching vibration of benzenoid ring and quinoid rings, respectively [2,3]. On the other hands, the spectral region between 1220 cm^{-1} and 1375 cm^{-1} contains signals attributed to different C-N vibrational modes, which are diagnostic of protonation degree.

In particular, the peaks around 1255 cm^{-1} and 1320 cm^{-1}, related to the stretching of semiquinone radicals $C\text{-}N^{+\bullet}$ polaronic band, arise from the protonated form of PANI [3,4]. Conversely, the band at 1225 cm^{-1} is ascribed to C-N stretching vibrations of the benzenoid configuration in deprotonated PANI units [5].

IR analysis

The FT-IR spectra shown in Figure S1 were acquired on the PANI (Fe) and PANIs (Fe) to study the chemical structure and to investigate the presence of sulfonate functionalities along PANIs backbone.

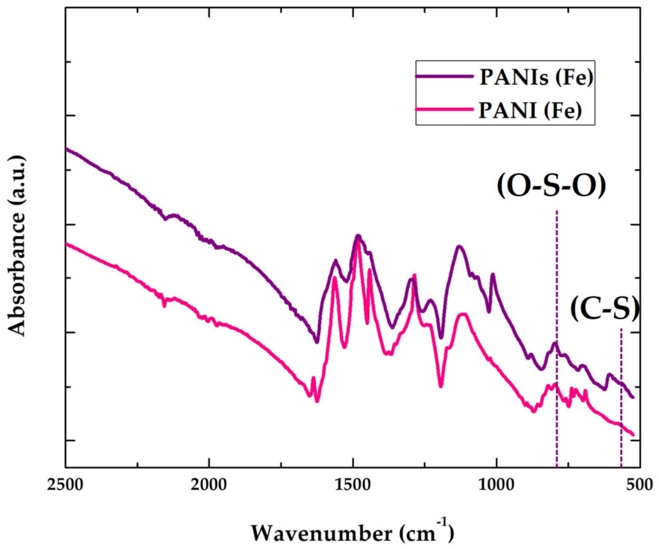

Figure S1. The IR spectra of PANI and PANIs.

As it can be seen from the Figure S1, both the PANI (Fe) polymer and the PANIS (Fe) copolymer have the characteristic peaks of PANI emeraldine salt.

In detail, the peaks around 1558 cm^{-1} and 1485 cm^{-1} are ascribed to the stretching vibration of quinoid and benzenoid rings. The PANIs (Fe) spectra shows two peaks at 1014 cm^{-1} and 608 cm^{-1} related to O-S-O and C-S stretching of the (SO3-) groups. These two peaks, completely absent in the PANI (Fe) spectrum, convincingly confirm the presence of sulfonated moieties along the PANIs backbone, due to the copolymerization of ANI and ANIs monomers.

Note S2: XRD analysis

As shown in **Figure 1c,** the XRD patterns of both PANI and PANIs exhibit the characteristic signals at about 15°, 20°, 25°, 27° and 29°, respectively corresponding to (001), (011), (020), (200), (121), and (022) diffraction planes of PANI emeraldine salt configuration [6,7]. In-depth structural details are attained by performing an X-ray Peak Profile Analysis (XPPA) of the polyaniline-related peaks of both the samples. In particular, the mean size of the polymeric crystallites (*d*) was calculated from position (2ϑ) and amplitude (FWHM) of the (200) reflections by means of the Scherrer formula [8].

The Scherrer formula used to calculate the averaged crystallites' dimensions is reported in Eq (S1):

$$d = \frac{0.89\ \lambda}{\beta \cos(\theta)} \qquad (S1)$$

where d represents the crystallite dimension, λ the specific X-ray wavelength, is the FWHM (in radians) of the deconvolved peak and is the diffraction angle.

EIS analysis

Table S1: EIS parameters for PEGDA-PANIs and PEGDA-PANI hydrogels.

Sample	R_s [Ω]	R_{ct} [Ω]	CPE		Z_W[kσ]
			Y_0 [μτ]	α [ϕ]	

PEGDA-PANIs	409	7200	3.4	0.815	6.1
PEGDA-PANI	440	3680	5.8	0.795	4.8

Table S2: Comparison of PANI sensors in terms of sensitivity, pH range analyzed and preparation methods.

Samples	pH range	Sensitivity (mV/pH)	Ref.
Chemical oxidative synthesis of PANI. Screen-printed electrode.	2-10	63.7	[9]
PANI electrochemical deposition on Interdigital gold electrode.	4-9	69.3	[10]
PANI Thin film electrochemical deposition on Graphene oxide.	2-9	55	[11]
Coaxial electrospun flexible PANI//PU fibers. PANI synthesis via the reverse microemulsion strategy. Screen-printed electrode.	2-7	60	[12]
Chemical oxidative synthesis of PANI. Screen printed electrode.	2-10	20.6	[13]
Chemical oxidative PANI synthesis. Spin coated interdigital electrode.	5.4-8.6	59.6	[14]
PANI electrodeposited on polymeric nanopillar arrays.	2-12	60.3	[15]

Printing apparatus

Figure S2 shows a picture of the ELEGOO Mars MSLA printer apparatus.

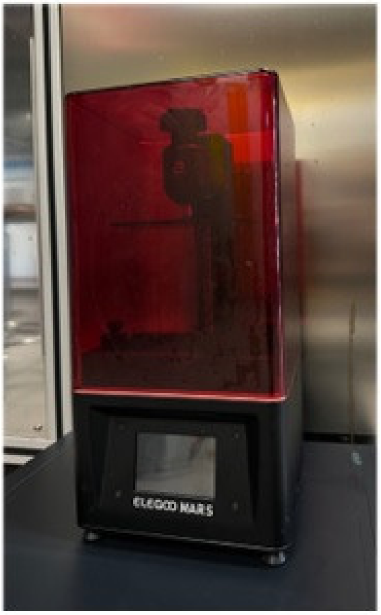

Figure S2. Picture of the ELEGOO Mars MSLA printer.

Samples' preparation

Table S3 Lists the lists the names of produced CPs reference samples, along with the concentrations of the monomers and reagents used to obtain the polymerizations' solutions.

Table S3: List of the experimental conditions for ANIs and ANI polymerization processes.

Sample name	Starting solution				Oxidant solution			Final solution	
	ANI (mmol)	ANIs (mmol)	DMSO (ml)	HCl (ml)	APS (mmol)	$FeCl_3$ (mmol)	HCl 1M (ml)	[ANI] (M)	[ANIs] (M)
PANIs	2	2	2	-	4	-	80	0.025	0.025
PANI	4	-	2	-	4	-	80	0.05	-
PANIs (Fe)	2	2	-	2	-	8	80	0.025	0.025
PANI (Fe)	4	-	-	2	-	8	80	0.05	-

Table S4 Lists the names of the produced 3D nanocomposites objects, along with the concentrations of the monomers and reagents used to obtain the polymerizations' solutions.

Table S4: List of experimental conditions for the in-situ polymerization processes.

Sample name	Soaking solution			Starting solution			APS solution		Final solution	
	ANI (mmol)	ANIs (mmol)	DMSO (ml)	ANI (mmol)	ANIs (mmol)	DMSO (ml)	APS (mmol)	HCl 1M (ml)	[ANI] (M)	[ANIs] (M)
PEGDA-PANIs	2	2	2	2	2	2	4	80	0.025	0.025

PEGDA-PANI	190	-	-	4	-	2	4	80	0.05	-

References

1. Suendo, V.; Lau, Y.; Hidayat, F.; Reza, M.; Qadafi, A.; Rochliadi, A. Effect of Face-to-Face and Side-to-Side Interchain Interactions on the Electron Transport in Emeraldine Salt Polyaniline. *Phys. Chem. Chem. Phys.* **2021**, *23*, 7190–7199, doi:10.1039/d0cp06194h.
2. Tamburri, E.; Guglielmotti, V.; Orlanducci, S.; Terranova, M.L.; Sordi, D.; Passeri, D.; Matassa, R.; Rossi, M. Nanodiamond-Mediated Crystallization in Fibers of PANI Nanocomposites Produced by Template-Free Polymerization: Conductive and Thermal Properties of the Fibrillar Networks. *Polymer (Guildf).* **2012**, *53*, 4045–4053, doi:10.1016/j.polymer.2012.07.014.
3. Lindfors, T.; Ivaska, A. Raman Based PH Measurements with Polyaniline. *J. Electroanal. Chem.* **2005**, *580*, 320–329, doi:10.1016/j.jelechem.2005.03.042.
4. Laska, J.; Girault, R.; Quillard, S.; Louarn, G.; Proń, A.; Lefrant, S. Raman Spectroscopic Studies of Polyaniline Protonation with Bis(2-Ethylhexyl) Hydrogen Phosphate. *Synth. Met.* **1995**, *75*, 69–74, doi:10.1016/0379-6779(95)03379-X.
5. Stejskal, J.; Trchová, M.; Bober, P.; Humpolíček, P.; Kašpárková, V.; Sapurina, I.; Shishov, M.A.; Varga, M. Conducting Polymers: Polyaniline. *Encycl. Polym. Sci. Technol.* **2015**, 1–44, doi:10.1002/0471440264.PST640.
6. Yang, S.; Zhu, S.; Hong, R. Graphene Oxide/Polyaniline Nanocomposites Used in Anticorrosive Coatings for Environmental Protection. *Coatings 2020, Vol. 10, Page 1215* **2020**, *10*, 1215, doi:10.3390/COATINGS10121215.
7. Sanches, E.A.; Soares, J.C.; Iost, R.M.; Marangoni, V.S.; Trovati, G.; Batista, T.; Mafud, A.C.; Zucolotto, V.; Mascarenhas, Y.P. Structural Characterization of Emeraldine-Salt Polyaniline/Gold Nanoparticles Complexes. *J. Nanomater.* **2011**, *2011*, 697071, doi:10.1155/2011/697071.
8. Wu, Q.; Xue, Z.; Qi, Z.; Wang, F. The Microscopic Morphology of Highly Sulfonated Polyaniline. *Synth. Met.* **2000**, *108*, 107–110, doi:10.1016/S0379-6779(99)00173-3.
9. Zhu, C.; Xue, H.; Zhao, H.; Fei, T.; Liu, S.; Chen, Q.; Gao, B.; Zhang, T. A Dual-Functional Polyaniline Film-Based Flexible Electrochemical Sensor for the Detection of PH and Lactate in Sweat of the Human Body. *Talanta* **2022**, *242*, 123289, doi:10.1016/j.talanta.2022.123289.
10. Zhao, Y.; Yu, Y.; Zhao, S.; Zhu, R.; Zhao, J.; Cui, G. Highly Sensitive PH Sensor Based on Flexible Polyaniline Matrix for Synchronal Sweat Monitoring. *Microchem. J.* **2023**, *185*, 108092, doi:10.1016/j.microc.2022.108092.
11. Chinnathambi, S.; Euverink, G.J.W. Polyaniline Functionalized Electrochemically Reduced Graphene Oxide Chemiresistive Sensor to Monitor the PH in Real Time during Microbial Fermentations. *Sensors Actuators, B Chem.* **2018**, *264*, 38–44, doi:10.1016/j.snb.2018.02.087.
12. Hou, X.; Zhou, Y.; Liu, Y.; Wang, L.; Wang, J. Coaxial Electrospun Flexible PANI//PU Fibers as Highly Sensitive PH Wearable Sensor. *J. Mater. Sci.* **2020**, *55*, 16033–16047, doi:10.1007/s10853-020-05110-7.
13. Bao, Q.; Yang, Z.; Song, Y.; Fan, M.; Pan, P.; Liu, J.; Liao, Z.; Wei, J. Printed Flexible Bifunctional Electrochemical Urea-PH Sensor Based on Multiwalled Carbon Nanotube/Polyaniline Electronic Ink. *J. Mater. Sci. Mater. Electron.* **2019**, *30*, 1751–1759, doi:10.1007/s10854-018-0447-\5.
14. Li, Y.; Mao, Y.; Xiao, C.; Xu, X.; Li, X. Flexible PH Sensor Based on a Conductive PANI Membrane for PH Monitoring. *RSC Adv.* **2019**, *10*, 21–28, doi:10.1039/c9ra09188b.
15. Yoon, J.H.; Hong, S.B.; Yun, S.O.; Lee, S.J.; Lee, T.J.; Lee, K.G.; Choi, B.G. High Performance Flexible PH Sensor Based on Polyaniline Nanopillar Array Electrode. *J. Colloid Interface Sci.* **2017**, *490*, 53–58, doi:10.1016/j.jcis.2016.11.033.
